# *Arcobacteraceae*: An Exploration of Antibiotic Resistance Featuring the Latest Research Updates

**DOI:** 10.3390/antibiotics13070669

**Published:** 2024-07-18

**Authors:** Davide Buzzanca, Elisabetta Chiarini, Valentina Alessandria

**Affiliations:** Department of Agricultural, Forest and Food Sciences, University of Turin, Largo Paolo Braccini nr.2, 10095 Grugliasco, Italy; elisabetta.chiarini@unito.it (E.C.); valentina.alessandria@unito.it (V.A.)

**Keywords:** *Arcobacter* spp., foodborne pathogen, zoonosis, antimicrobial resistance, multiple drug resistance, food safety

## Abstract

The *Arcobacteraceae* bacterial family includes species isolated from animals and related food products. Moreover, these species have been found in other ecological niches, including water. Some species, particularly *Arcobacter butzleri* and *Arcobacter cryaerophilus*, have been isolated from human clinical cases and linked to gastrointestinal symptoms. The presence of antibiotic-resistant strains is a concern for public health, considering the possible zoonoses and foodborne infections caused by contaminated food containing bacteria resistant to antibiotic treatments. This review aims to highlight the importance of antibiotic resistance in *Arcobacter* spp. isolates from several sources, including information about antibiotic classes to which this bacterium has shown resistance. *Arcobacter* spp. demonstrated a wide spectrum of antibiotic resistance, including several antibiotic resistance genes. Antibiotic resistance genomic traits include efflux pumps and mutations in antibiotic target proteins. The literature shows a high proportion of *Arcobacter* spp. that are multidrug-resistant. However, studies in the literature have primarily focused on the evaluation of antibiotic resistance in *A. butzleri* and *A. cryaerophilus*, as these species are frequently isolated from various sources. These aspects underline the necessity of studies focused on several *Arcobacter* species that could potentially be isolated from several sources.

## 1. Introduction

The *Arcobacteraceae* bacterial family includes Gram-negative species isolated from several environment matrices and hosts. Some of these species have been isolated from animals in which these bacteria have shown pathogenicity. Recently, a division between pathogenic and non-pathogenic strains has been proposed [1]. *Arcobacter butzleri* and *Arcobacter cryaerophilus* are considered the two species in the *Arcobacteraceae* family that are most frequently associated with clinical outbreaks. Although to a lesser extent, *Arcobacter cibarius*, *Arcobacter thereius*, and *Arcobacter skirrowii* are also considered pathogens. The main symptoms of *Arcobacter* spp. infection are related to gastrointestinal disorders, with diarrhoea being the most prominent. *Arcobacter* spp. is widely considered a zoonotic pathogen related to foodborne diseases. Furthermore, it is important to consider that *Arcobacter* spp. can be mistaken for *Campylobacter* spp. during clinical analyses, warranting additional attention to this pathogen [2]. The species included in the *Arcobacteraceae* family usually do not cause symptoms in animals [3]. The asymptomatic behaviour of these bacteria can increase their spread, making them more difficult to identify directly. Although *Arcobacter* infection often remains asymptomatic, these bacteria have been associated with various symptoms in some cases. *A. butzleri* has been linked to enteritis, with symptoms of diarrhoea in cattle, pigs, and horses [4,5]. *A. butzleri* has also been isolated from faecal samples of chickens, turkeys, ducks, and domestic geese [6,7]. In research performed in Türkiye, *A. butzleri* was the species most frequently isolated from chickens, geese, ducks, turkeys, and quails, followed by *A. cryaerophilus*, *A. skirrowii*, and *A. cibarius* [8]. The species *A. thereius* has been isolated from pigs and ducks in Belgium [9]. *A. skirrowii* has been associated with diarrhoea and haemorrhagic colitis in cattle and sheep [5,10].

The isolation of *Arcobacter* spp. from animals can be linked to its presence in food [11]. Food is considered one of the main transmission sources of *Arcobacter* spp., which, due to their pathogenicity, are considered foodborne pathogens. The principal foods found to be contaminated by *Arcobacter* spp. are of animal origins (clams, milk, meat, and fish), with chicken meat showing a high percentage of isolation related to this bacterial genus. However, *Arcobacter* spp. have also been found in vegetables and ready-to-eat vegetables. Regarding vegetables, *Arcobacter* spp. have been detected on lettuce [12], rocket [13], napa cabbage, water parsley [14], and ready-to-eat salad [15]. The species most frequently isolated from vegetables is *A. butzleri*, while *A. cryaerophilus* has been isolated from leafy green vegetables [14]. *A. butzleri* can survive in the apple and pear puree production process, although with a significant reduction in the bacterial load [16].

Considering the isolation of *Arcobacter* spp. from clinical cases and foods, and its pathogenicity in vitro, antibiotic-resistant strains represent a risk to public health. This aspect is related to the loss of antibiotic efficacy in case of infections [17]. Moreover, the possible horizontal gene transfer of antibiotic resistance genes to other bacteria cannot be excluded. *Arcobacter* spp. represents a widely distributed human pathogen among foods, water, animals, and environmental niches [1]. The antimicrobial resistance (AMR) of *Arcobacter* spp. underlines its importance as a pathogen due to the possible risk of infection after contact with contaminated sources treatable with reduced effectiveness due to decreased or absent effects of antibiotics. This review will discuss the AMR of *Arcobacter* spp., highlighting observations related to antibiotic-resistant strains from different sources, including food, water, and animals. Information about the mechanisms of *Arcobacter* spp. of antibiotic resistance mechanisms will also be discussed. This review aims to highlight the antibiotic resistance of *Arcobacter* spp., focusing on pathogenic species in humans with data from recent studies.

## 2. Antibiotic Resistance of *Arcobacter* Spp. Isolated from Food and Related Land Animals

*Arcobacter* spp. strains, isolated from foods, are resistant to several antibiotic classes (Table 1). The AMR of *Arcobacter* spp. has been demonstrated for different species isolated from various sources. Specific protocols for the AMR resistance evaluation of *Arcobacter* spp. are not available in the official guidelines [18]. For this reason, breakpoint and method references will be indicated in the text considering the different methods used in the AMR determination of *Arcobacter* spp. These specifications are rendered necessary because different AMR evaluation methods may lead to different results [19,20]. In Portugal, *Arcobacter* spp. showed multidrug resistance (MDR) following the antibiotic dilution method (European Committee on Antimicrobial Susceptibility Testing; EUCAST breakpoints) in 85.7% of the isolates from food samples [21]. These authors demonstrated a high AMR in *A. butzleri* and *A. cryaerophilus*, especially to nalidixic acid (100% of *A. butzleri* and 87.5% of *A. cryaerophilus* isolates), tetracycline (95.4% of *A. butzleri* and 93.8% of *A. cryaerophilus* isolates), and cefotaxime (98.5% of *A. butzleri* and 93.8% of *A. cryaerophilus* isolates), while gentamicin was effective against all isolates [21]. High resistance of *A. butzleri* to nalidixic acid (agar dilution method; Centers for Disease Control and Prevention; CDS and Clinical & Laboratory Standards Institute; CLSI break points) was confirmed by Isidro and colleagues in 22 strains from poultry samples, meats and vegetables, raw milks, and from a dairy plant environment, resulting instead in susceptibility to gentamicin [22]. Resistance to cefoperazone (disk diffusion method; CLSI breakpoints) has been demonstrated for *A. butzleri*, *A. cryaerophilus*, and *A. skirrowii* isolated from meat [23]. The evaluation of AMR in *Arcobacter* spp. from several sources (poultry meat, patients, and water) following a disk diffusion method (CLSI breakpoints) shows that most isolates were resistant to β-lactam antibiotics [24]. In the case of poultry meat, *A. butzleri* was found to be antibiotic-resistant to ampicillin, aztreonam, cephalothin, clindamycin, nalidixic acid, oxacillin, and penicillin G, while *A. cryaerophilus* isolates were resistant to clindamycin, oxacillin, and penicillin G [24]. Ampicillin, erythromycin, and tetracycline showed low efficacy against *A. butzleri* from chicken and cattle meat after a disk diffusion method evaluation (EUCAST breakpoints) [25]. *A. skirrowii* isolated from poultry water was found to be resistant to streptomycin following a gradient strip diffusion method (E-test; EUCAST breakpoints) [26]. *A. butzleri*, *A. cryaerophilus*, and *A. skirrowii* from chicken samples in Egypt showed resistance (disk diffusion method; CLSI breakpoints) against ampicillin, ampicillin-sulbactam, and cefotaxime [27]. Although the literature primarily focused on isolates from poultry meat, cases of AMR have been observed for *Arcobacter* spp. isolated from other meats. A high number of isolates resistant to cefotaxime, nalidixic acid, and tetracycline was observed for *Arcobacter* spp. isolates from pork and beef meat (antibiotic dilutions method; EUCAST breakpoints) [21]. A study on *A. butzleri* from fresh raw cattle meat samples showed AMR (disk diffusion method; CLSI breakpoints) to tetracycline (72%), amoxicillin (69%), erythromycin (67%), and cefoxitin (66%), while 60% of *A. cryaerophilus* isolates were resistant to cefoxitin and erythromycin, confirming MDR phenomena in these species [28]. Other important foods of animal origin in which *Arcobacter* spp. has been isolated are milk and dairy products. A study conducted in Iran demonstrated resistance to amoxicillin–clavulanic acid and tetracycline in *A. butzleri* isolated from milk, with some cases of AMR (disk diffusion method) for *A. cryaerophilus* isolates from the same matrix [29]. *A. butzleri* and *A. cryaerophilus* isolated from milk were found to be resistant to amoxicillin–clavulanic acid and tetracycline [29]. Strains of *A. butzleri* isolated from chicken breast and fresh vegetables demonstrate MDR (disk diffusion method; EUCAST breakpoints) to tetracyclines and cefotaxime (third-generation antibiotic) [30].

*Arcobacter* spp. have been isolated from several land animals, including farm animals. However, most research on AMR in *Arcobacter* spp. has been focused on animal products such as milk and meat; for this reason, only a few recent works are mentioned here. *A. butzleri* isolates from healthy pigs’ faecal samples (*n* = 203) showed resistance (disk diffusion method, CLSI) to cefotaxime in 98.6% of isolates, and 71% of isolates showed resistance to sulbactam–cefoperazone followed by ampicillin (67.7%), while AMR to enrofloxacin (48.4%) and fosfomycin (42.9%) was lower [31]. *Arcobacter* spp., with a prevalence of *A. butzleri*, isolated from pigs, ducks, quails, and sheep in Ghana and Tanzania showed a 100% antibiotic resistance rate to ampicillin, chloramphenicol, and penicillin (disk diffusion method, EUCAST) [32].

The ability of *Arcobacter* spp. to colonize several surfaces has also been demonstrated [33,34]. Some recent studies on *Arcobacter* spp. isolated from food processing plant surfaces are present in the literature. *A. butzleri* isolated from a dairy plant in Portugal showed resistance to nalidixic acid and susceptibility to erythromycin and gentamicin [31]. However, isolates from slaughterhouse surfaces, even when showing resistance to ampicillin and nalidixic acid, also demonstrated resistance to erythromycin, indicating variable results between isolates from different sources [35]. *A. butzleri* strains from a chicken slaughterhouse in Italy (chicken skins, cloacae, and surfaces) [36] demonstrated MDR (agar diffusion method, EUCAST breakpoints) to amoxicillin–clavulanic acid, amoxicillin, ampicillin, azithromycin, clarithromycin, erythromycin, and gentamicin [37].

The wide prevalence of antibiotic-resistant *Arcobacter* spp. strains in food and production plants, in addition to their pathogenic potential, underlines their dangers as food contaminants. This is even more evident considering that antibiotic resistance leads to a loss of antibiotic efficacy, resulting in difficulties in treating bacterial infections [16].

**Table 1:** Species of *Arcobacter* spp. showing AMR to several antibiotic classes, isolated from meat, food, and related animals. The table indicates antibiotics, their classes, and the sources from which *Arcobacter* spp. showed resistance.

Species	Antibiotic	Class	Sources	Refs.
*A. butzleri* and *A. cryaerophilus*	Nalidixic acid	Quinolone	Meat and related animals	[21,22,24]
*A. butzleri*, *A. cryaerophilus*, and *A. skirrowii*	Cefotaxime	Cephalosporin	Meat and related animals	[21,27]
*A. butzleri, A. cryaerophilus*, and *A. skirrowii*	Cefoperazone	Cephalosporin	Meat and related animals	[23]
*A. butzleri*, *A. cryaerophilus*, and *A. skirrowii*	Ampicillin	Penicillin	Meat and related animals	[24,25,27]
*A. butzleri*	Aztreonam	Monobactams	Meat and related animals	[24]
*A. butzleri*	Cephalothin	Cephalosporin	Meat and related animals	[24]
*A. butzleri* and *A. cryaerophilus*	Clindamycin	Lincosamide	Meat and related animals	[24]
*A. butzleri* and *A. cryaerophilus*	Oxacillin	Penicillin	Meat and related animals	[24]
*A. butzleri* and *A. cryaerophilus*	Penicillin G	Penicillin	Meat and related animals	[24]
*A. butzleri*	Erythromycin	Macrolide	Meat and related animals, food processing plant surfaces	[25,28,35,37]
*A. butzleri* and *A. cryaerophilus*	Tetracycline	Tetracycline	Meat and related animals	[21,25,28]
*A. butzleri*	Amoxicillin	Penicillin	Meat and related animals, food processing plant surfaces	[28,37]
*A. butzleri* and *A. cryaerophilus*	Cefoxitin	Cephamycin	Meat and related animals	[28]
*A. butzleri*, *A. cryaerophilus*, and *A. skirrowii*	Ampicillin–sulbactam	Penicillin and beta-lactamase inhibitors	Meat and related animals	[27]
*A. butzleri*	Amoxicillin–clavulanic acid	Penicillin and beta-lactamase inhibitors	Milk, dairy products, meat and related animals, food processing plant surfaces	[29,37]
*A. butzleri*	Tetracycline	Tetracycline	Milk and dairy products, meat and related animals, fresh vegetables	[29,30]
*A. butzleri*	Nalidixic acid	Quinolone	Food processing plant surfaces	[35,38]
*A. butzleri*	Ampicillin	Penicillin	Meat and related animals, food processing plant surfaces, pigs, ducks, quails, and sheep	[31,32,35,37]
*A. butzleri*	Azithromycin	Macrolide	Meat and related animals, food processing plant surfaces	[37]
*A. butzleri*	Clarithromycin	Macrolide	Meat and related animals, food processing plant surfaces	[37]
*A. butzleri*	Gentamicin	Aminoglycoside	Meat and related animals, food processing plant surfaces	[37]
*A. butzleri*	Cefotaxime	Cephalosporin	Meat and related animals, fresh vegetables	[30]
*A. butzleri*	Cefoperazone–sulbactam	Cephalosporin and beta-lactamase inhibitors	Pigs	[31]
*A. butzleri*	Chloramphenicol	Amphenicol	Pigs, ducks, quails, and sheep	[32]
*A. butzleri*	Penicillin	Penicillin	Pigs, ducks, quails, and sheep	[32]

## 3. Antibiotic Resistance of *Arcobacter* Spp. Isolated from Water and Water Animals

*Arcobacter* spp., and in particular *A. butzleri,* isolated from water and water animals demonstrated resistance to several classes of antibiotics (Table 2). *Arcobacter* spp. has been positively correlated with the antibiotic’s presence in river water [39]. Cases of resistance to high concentrations of ampicillin (>256 µg/mL), azithromycin (>256 µg/mL), and ciprofloxacin (>32 µg/mL) were observed in *A. butzleri* isolated from surface waters, including river and lake water [15]. Twenty-seven *A. butzleri* isolates recovered from aquatic environments were resistant to ampicillin, cephalothin, cefotaxime, nalidixic acid, and tetracycline (disk diffusion method, CLSI) [40]. The resistance to cefotaxime, a third-generation antibiotic, demonstrated in *A. butzleri* underlines the ability of this bacterium to withstand new antimicrobial molecules. *A. butzleri* and *A. cryaerophilus* isolated from water showed MDR in 94.4% and 66.7% of the strains tested, respectively (disk diffusion method, CLSI) [24]. *A. butzleri* isolated from wastewater showed MDR to aztreonam, ampicillin, cephalothin, clindamycin, nalidixic acid, oxacillin, and penicillin G [24]. *A. butzleri* was found in agricultural surface water (913 isolates) demonstrating, in most cases, resistance against clindamycin (99%) and chloramphenicol (77%) (agar dilution method, CLSI) [41].

As stated, *Arcobacter* spp. has been isolated from water animals and related food products. AMR tests were performed on these isolates. Strains of *A. butzleri* isolated from sushi showed MDR (disk diffusion method, EUCAST) to tetracyclines and cefotaxime [30]. A study conducted in Italy showed the presence of AMR *Arcobacter* strains in mussels and clams from a local fish market (disk diffusion method, CLSI) [42]. Two strains showed high resistance to β-lactams (ampicillin, penicillin, and cefotaxime) as well as tetracycline, and erythromycin [42]. Other authors demonstrated a high AMR (disk diffusion method, CLSI) of *A. butzleri* isolated from seafood to cephalothin, cefoxitin, and sulfamethizole [43]. *Arcobacter* spp. was isolated from catla (*Catla catla*) samples from markets and aquaculture ponds, demonstrating MDR (disk diffusion method, CLSI) in five isolates of *A. butzleri* [44]. Three of these isolates showed resistance to penicillin and cefixime, while two isolates showed resistance to penicillin, nalidixic acid, and erythromycin [44]. *A. butzleri* strains from clams (*Tapes philippinarumand*) and mussels (*Mytilus galloprovincialis*) were found to be resistant to ampicillin, penicillin, cefotaxime, tetracycline, and erythromycin, while one strain was resistant to nalidixic acid (disk diffusion method, CLSI) [42].

The widespread presence of *Arcobacter* spp. in water and water animals and their AMR draws attention to the risk associated with ingesting antimicrobial-resistant strains from these sources.

**Table 2:** AMR of *A. butzleri* isolated from water, water environments, and related animals and food. The table indicates antibiotics, their classes, and the sources of isolation from which *A. butzleri* showed resistance.

Antibiotic	Class	Sources	Refs.
Ampicillin	Penicillin	Surface water, aquatic environments, wastewater, mussels and clams	[15,24,40,42]
Azithromycin	Macrolide	Surface water	[15]
Ciprofloxacin	Fluoroquinolone	Surface water	[15]
Cephalothin	Cephalosporin	Aquatic environments, wastewater, seafood	[24,40,43]
Cefotaxime	Cephalosporin	Aquatic environments	[40]
Nalidixic acid	Quinolone	Aquatic environments, wastewater, *Catla catla*	[24,40,44]
Tetracycline	Tetracycline	Aquatic environments, sushi, mussels and clams	[30,40,42]
Aztreonam	Monobactam	Wastewater	[24]
Clindamycin	Lincomycin	Wastewater	[24]
Oxacillin	Penicillin	Wastewater	[24]
Penicillin G	Penicillin	Wastewater	[24]
Clindamycin	Lincosamide	Agricultural surface water	[41]
Chloramphenicol	Amphenicol	Agricultural surface water	[41]
Cefotaxime	Cephalosporin	Sushi, mussels and clams	[30,42]
Penicillin	Penicillin	Mussels and clams, *Catla catla*	[42,44]
Erythromycin	Macrolide	Mussels and clams, *Catla catla*	[42,44]
Cefoxitin	Cephalosporin	Seafood	[43]
Sulphamethizole	Sulfonamide	Seafood	[43]
Cefixime	Cephalosporin	*Catla catla*	[44]

## 4. Antibiotic Resistance of *Arcobacter* Spp. Isolated from Humans

Species of *Arcobacter* spp., prevalently *A. butzleri* and *A. cryaerophilus*, have been isolated from human clinical cases (Table 3). Clinical cases related to *Arcobacter* spp. are normally solved without the need for antibiotic treatment [45]. However, in some cases, treatment has been necessary. A study that included samples from German patients from whom *A. butzleri*, *A. cryaerophilus*, and *Arcobacter lanthieri* had been isolated demonstrated that ciprofloxacin (E-test; CLSI) was the most appropriate antibiotic among those tested [46]. An *Arcobacter* spp. infection in a COVID-19 and HIV patient was resolved with a treatment that included intravenous meropenem for five days followed by oral ciprofloxacin [47]. *A. lanthieri* was isolated in Belgium from a patient with abdominal bloating and cramps [48]. In this case, the infection resolved spontaneously, but the isolate showed AMR (E-test; EUCAST) to ampicillin, ciprofloxacin, and erythromycin [48].

The in vitro AMR of *Arcobacter* spp. isolated from clinical samples has been observed. *A. butzleri* and *A. cryaerophilus* isolated from Belgian patients were found to be resistant (E-test; EUCAST) to ampicillin (91% of the strains) [49]. A study conducted in *A. butzleri* isolates from clinical samples showed high AMR (E-test; CLSI) to ampicillin (MIC; 24–64 µg/mL) [46]. Two *A. butzleri* strains isolated from a patient with travellers’ diarrhoea and from another with pruritus showed AMR to tetracycline, while amoxicillin–clavulanic acid and ampicillin AMRs (MIC test strip; EUCAST) were observed in one strain [50]. A study performed in Central Italy demonstrated AMR in an *A. butzleri* strain to amoxicillin–clavulanic acid, ampicillin, tetracycline, ciprofloxacin, nalidixic acid, cefalotin, cefotaxime, erythromycin, gentamicin, and streptomycin (disk diffusion test; EUCAST and CLSI) [30]. Another strain from the same study was susceptible to amoxicillin–clavulanic acid and showed intermediate resistance to gentamicin [30]. Šilha and colleagues observed a high AMR ratio in *A. butzleri* from human enteritis cases, with at least six of the seven strains tested resistant to ampicillin, aztreonam, chloramphenicol, clindamycin, nalidixic acid, oxacillin, and penicillin G (disk diffusion test; CLSI) [24]. All *A. butzleri*, *A. cryaerophilus*, and *A. skirrowii* isolated in a study conducted in Iran demonstrated AMR against cefazolin, ceftazidime, and nalidixic acid (disk diffusion test; CLSI) [51]. Moreover, all *A. butzleri* isolates demonstrated AMR to chloramphenicol [51].

The AMR assays demonstrate that *Arcobacter* spp. show resistance to several antibiotic classes even in isolates from human clinical cases. This aspect underlines the importance of *Arcobacter* spp. as a bacterial pathogen.

**Table 3:** Species of *Arcobacter* spp. showing AMR to several antibiotic classes, isolated from clinical cases. The table indicates antibiotics, their classes, and the sources from which *Arcobacter* spp. showed resistance. Literature references are included in the last columns.

Species	Antibiotic	Class	Refs.
*A. butzleri* and *A. cryaerophilus*	Ampicillin	Penicillin	[24,30,46,49,50]
*A. butzleri*	Amoxicillin–clavulanic acid	Penicillin and beta-lactamase inhibitors	[30,50]
*A. butzleri*	Aztreonam	Beta-lactam	[24]
*A. butzleri*, *A. cryaerophilus*, and *A. skirrowii*	Cefalotin	Cephalosporin	[30,51]
*A. butzleri*, *A. cryaerophilus*, and *A. skirrowii*	Cefazolin	Cephalosporin	[51]
*A. butzleri*	Cefotaxime	Cephalosporin	[30]
*A. butzleri*, *A. cryaerophilus*, and *A. skirrowii*	Ceftazidime	Cephalosporin	[51]
*A. butzleri*	Chloramphenicol	Amphenicol	[24,51]
*A. butzleri*	Ciprofloxacin	Fluoroquinolone	[30]
*A. butzleri*	Clindamycin	Lincomycin	[24]
*A. butzleri*	Erythromycin	Macrolide	[30]
*A. butzleri*	Gentamicin	Aminoglycoside	[30]
*A. butzleri*, *A. cryaerophilus*, and *A. skirrowii*	Nalidixic acid	Quinolone	[24,30,51]
*A. butzleri*	Oxacillin	Penicillin	[24]
*A. butzleri*	Penicillin G	Penicillin	[24]
*A. butzleri*	Streptomycin	Aminoglycoside	[30]
*A. butzleri*	Tetracycline	Tetracycline	[30,50]

## 5. Genomic Traits Related to Antibiotic Resistance

**Figure 1:**
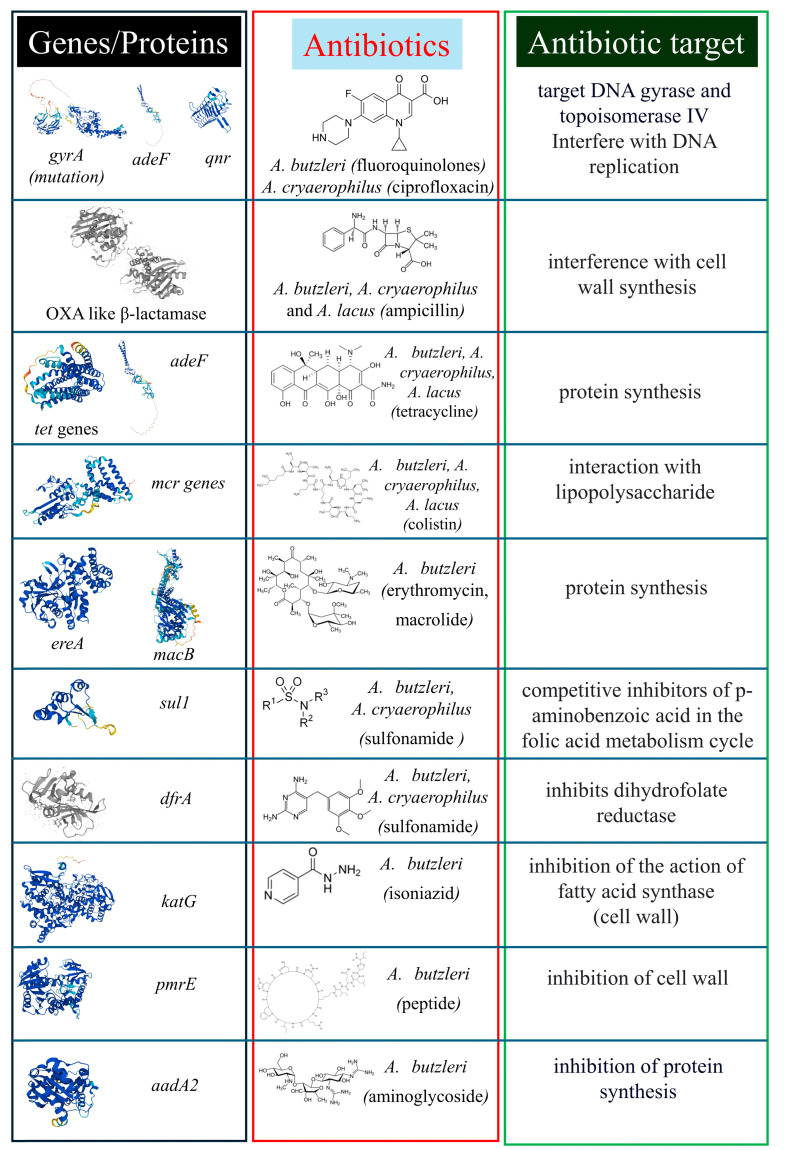
AMR mechanisms in *Arcobacter* spp. The figure shows genomic traits at which *Arcobacter* spp. resulted in antibiotic resistance or that were detected through molecular methods. Antibiotics/classes and related mechanisms of action are included in the red and green boxes. The protein figures were uploaded from Uniprot (https://www.uniprot.org/; accessed on 7 June 2024) [57].

The high AMR of *Arcobacter* spp. suggests the presence of genomic determinants in its genome (Figure 1). The antibiotic resistance of *Arcobacter* spp. has been correlated to specific genetic factors. Isidro and colleagues linked the AMR (agar dilutions method; CLSI breakpoints) of *A. butzleri* to fluoroquinolones with Thr-85-Ile in GyrA, while ampicillin resistance was associated to OXA-15-like β-lactamase [22]. Similarly, *A. cryaerophilus* isolated from water poultry and resistant (E-test; EUCAST breakpoints) to ciprofloxacin showed a point mutation (Thr-85-Ile) in *gyrA* [52]. *A. butzleri* and *A. cryaerophilus* isolated from water sources presented *tetW* (tetracycline resistance), while *A. butzleri* was also characterized by *tetO* and *tetA* [40]. A study conducted on antibiotic-resistant *A. butzleri* isolates (disk diffusion method; CLSI breakpoint) from shellfish determined the presence of DegT/DnrJ/EryC1/StrS aminotransferase family protein, which is required for the resistance to polymyxin and cationic antimicrobial peptides and HipA (type II toxin-antitoxin system) involved in methicillin resistance [42]. The same authors detected the presence of outer membrane efflux protein-related genes linked to AMR; among these were the genes *feoA* and *feoB* [42]. Antibiotic resistance genes *blaOXA-61*, *tetO,* and *tetW* were found in all *A. butzleri*, *A. cryaerophilus*, and *A. lacus* isolates obtained from seafood and water samples [53]. Colistin resistance genes (*mcr1/2/6*, *mcr3/7*, *mcr4*, *mcr5*, and *mcr8*) where found in part of the isolates, with *mcr5* present in all *A. cryaerophilus* isolates [53].

A study conducted in China demonstrated that *A. butzleri* and *A. cryaerophilus* isolated from pork and chicken harboured resistance island gene clusters, while an *A. butzleri* isolate showed *ereA*, a macrolide resistance gene [54]. *A. butzleri* and *A. cryaerophilus* isolated from cattle meat demonstrated the presence of the AMR genes *qnr* (quinolone resistance gene), *dfrA1* (dihydrofolate reductase), *tetB* and *tetA* (tetracycline resistance), *blaCITM* and *blaSHV* (beta-lactam resistance), and *sul1* (sulfonamide resistance) [28]. Genomes of *A. butzleri* isolates from human clinical cases contained *tetO,* linked to tetracycline resistance, and *bla3,* linked to ampicillin and amoxicillin–clavulanic resistance [50]. The presence of AMR genes revealed its influence on the antibiotic resistance of *Arcobacter* to several antibiotics. Strains of *A. butzleri* isolated from cow milk harboured the *adeF* gene (present in all strains, conferring resistance to fluoroquinolone and tetracycline), while 90% of the strains harboured the *acrB* gene (conferring resistance to rifamycin, cephalosporin, triclosan, glycylcycline, tetracycline, penam, phenicol, and fluoroquinolone) [55]. Some 30% of strains demonstrated the presence of *pmrE* (conferring resistance to polypeptide antibiotics), while 10% of strains carried *aadA2* (aminoglycoside resistance) and *macB* (macrolide resistance) [55]. Additionally, in this work, the mutations S140N, A139V, R463L, and A379T of the *katG* gene, conferring resistance to isoniazid, were detected in 50% of the strains [55].

Similarly to the mentioned *gyrA*, genetic variants and orthologues can differentially influence antibiotic resistance. A study conducted on 31 *A. butzleri* strains isolated from chicken carcasses and slaughterhouse equipment demonstrated a correlation between *hlyD* orthologues and AMR to several antibiotics (agar diffusion method, EUCAST breakpoints) [37]. The same pangenome study demonstrated a correlation of RND efflux pump and hydrophobe/amphiphile efflux-1 with AMR and a correlation of *mexAB-oprM* operon and *cydB* with MDR [37]. Another study on *A. butzleri* isolates from poultry suggested the importance of *oxa-464* and T81I point mutations in the quinolone resistance-determining region (disk diffusion method; EUCAST and CLSI breakpoints) [56].

## 6. Conclusions

*Arcobacter* spp. is considered an emergent foodborne pathogen, characterized by high persistence in food production plants [37]. Moreover, *Arcobacter* spp.’s presence in animals is well known [3]. For these reasons, the emergence of resistance to several antibiotic classes is considered an additional public health risk due to clinical treatment ineffectiveness (Figure 1, Table 1, Table 2 and Table 3) [58]. As stated, recent studies highlighted the MDR of *Arcobacter,* spp. including to several classes. *Arcobacter* spp. demonstrated a wide range of AMR traits (Figure 1). This can be linked to the presence of efflux pumps that can confer AMR to a wide range of antibiotics and to specific AMR genes. However, the high presence of hypothetical proteins in *Arcobacter* spp. [1] limits a comprehensive genome exploration linked to AMR. Even if procedures recommended by the Clinical and Laboratory Standards Institute and the European Committee on Antimicrobial Susceptibility Testing for *Campylobacter* or *Enterobacterales* are normally used for *Arcobacter* spp., the absence of standard procedures in AMR determination [18] can lead to different results between authors. This suggests the necessity of including *Arcobacter* spp. in official internationally recognized procedures. The current knowledge about *Arcobacter* spp. AMR is principally focused on *A. butzleri*, followed by *A. cryaerophilus*. Moreover, the number of studies focused on clinical isolates is low compared to food-related studies. Further studies are needed to increase the knowledge about AMR in this bacterial genus, including additional species and isolation sources. Moreover, an approach based on genomic analysis to be correlated to in vitro antibiotic studies and gene transformation of possible candidate resistance genes will allow for more precise identification of genetic traits linked to antibiotic resistance. This will enable the design of new analytical methods for the detection of *Arcobacter* spp. resistant to antibiotics.
